# Longitudinal changes in income are associated with the healthiness and sustainability of foods purchased in Mexican households

**DOI:** 10.1017/S1368980025100700

**Published:** 2025-07-17

**Authors:** Carolina Batis, Analí Castellanos-Gutiérrez, M Arantxa Colchero, Juan A. Rivera

**Affiliations:** 1 Nutrition and Health Research Center, National Institute of Public Health, Cuernavaca, Morelos, Mexico; 2 Harvard T.H. Chan School of Public Health, Harvard University, Boston, MA, USA; 3 Center for Health Systems Research, National Institute of Public Health, Cuernavaca, Morelos, Mexico; 4 Population Health Research Center, National Institute of Public Health, Cuernavaca, Morelos, Mexico

**Keywords:** Mexico, Food purchases, Income, Healthy, Sustainable

## Abstract

**Objective:**

To estimate the within-households association between change in income over time and food purchases in a national panel of households. The need to shift towards healthy and sustainable diets is widely recognised, thus the importance of identifying the factors that influence food purchase decisions.

**Design:**

Longitudinal observational study; for each of the thirty-three food items queried, we ran a conditional logistic fixed-effect regression model to evaluate the association between change in income per-capita and food purchases (yes/no) during the past week, adjusted by covariates.

**Setting:**

Mexican Family Life Survey.

**Participants:**

6008 households that participated in the survey for at least two of the three available waves of study (2002, 2005 and 2009).

**Results:**

Within-households, the OR (95 % CI) of purchasing the food in the past week for an increase in 1 sd of income was 1·09 (1·02, 1·16) for rarer fruits (other than bananas, apples and oranges); 1·11 (1·04, 1·18) for beef; 1·06 (1·00, 1·13) for canned tuna/sardines; 1·09 (1·02, 1·18) for fish/shellfish; 1·08 (1·02, 1·16) for discretionary packaged products and 1·15 (1·08, 1·23) for soft drinks. There were some differences by urban/rural area or socio-economic status (SES); mainly, those with lower SES had increased odds of purchasing the food item in more cases (ten out of thirty-three food items).

**Conclusions:**

Households’ income growth can have mixed effects on the healthiness and sustainability of food purchases. Public policies to improve the food environment and nutrition education are necessary to enhance the positive and counteract the negative effect of income.

## Graphical abstract

**Figure:**
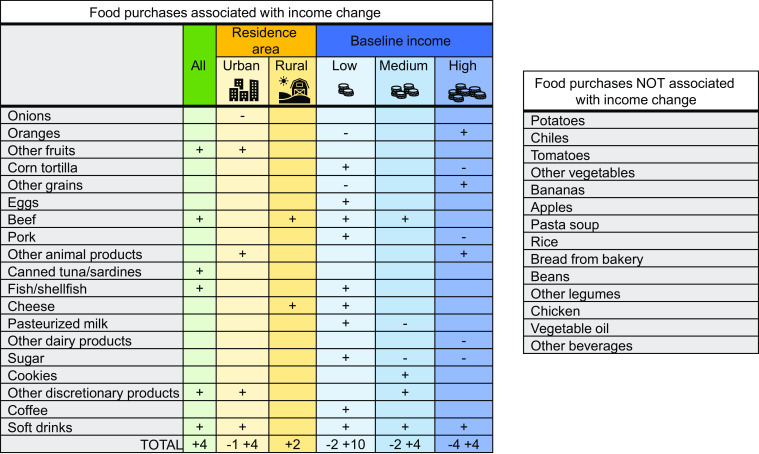

The Mexican population is facing an enormous public health challenge with its current prevalence of obesity and related co-morbidities. A third of children and adolescents and two-thirds of adults have overweight or obesity^(1)^. The associated loss in quality of life and economic costs are very serious. In 2014, it was estimated that the cost of obesity was $150 000 million Mexican pesos in direct health services costs and $70 000 million in indirect costs related to loss of productivity^(2)^. Dietary intake is a key factor in the development or prevention of these diseases. In Mexico, 34 % and 49 % of deaths caused by diabetes and heart diseases, respectively, are attributable to poor dietary habits^(3)^. Furthermore, global agricultural production is a leading cause of environmental degradation, is the main driver of biodiversity loss, land conversion, and freshwater use, and contributes significantly to climate change generating 21–37 % of greenhouse gas emissions in the world^(4)^. Hence, there is an urgent need to change towards healthy and sustainable diets that address simultaneously human and planetary health. Healthy and sustainable diets should be based on large amounts of vegetables, fruits, whole grains, legumes, nuts and unsaturated oils; moderate amounts of seafood, poultry and eggs and low or no amounts of red meat, processed meats, added sugar, refined grains and ultra-processed foods^(5–7)^.

To tackle the burden of poor dietary habits on the health of the population and the planet, it is necessary to understand the drivers behind individual food choices. Food selection is influenced by many factors such as culture, preferences, food availability, knowledge and beliefs. A factor particularly important is the price of food and related to this also is the economic income of the individuals^(8)^. In developed countries, it has been reported that healthy diets are more expensive than unhealthy ones; and that people with higher socio-economic status (SES) eat healthier^(8–11)^. In Mexico, we have reported that healthy diet baskets are not more expensive than current, less healthy ones and that healthy and sustainable baskets with fewer animal sources are even cheaper^(12)^. We also have documented that in Mexico SES has mixed associations with the healthfulness of diets. Individuals with higher SES not only consume more fruits, vegetables and dairy but also consume more processed discretionary foods (i.e. junk food), red and processed meats and fewer legumes, whereas the intake of sugar-sweetened beverages is similar across SES levels^(13)^. Events like the COVID-19 pandemic that caused a great economic disruption, particularly in the most vulnerable populations^(14)^, highlight the relevance of understanding how income affects food purchasing behaviors and how this differs across subpopulations.

Most of the evidence on the association between SES and diet quality comes from cross-sectional data. However, SES or income level might be associated with other factors such as the neighbourhood’s food environment, knowledge, beliefs, personal preferences or traditions. Therefore, to better understand the effect of income, we need to analyse a longitudinal study. Fixed-effects models are based on the variation over repeated measurements within-units of analysis, meaning that time-invariant unmeasured or unobserved confounding factors are controlled for. There is very limited evidence worldwide on how changes in incomes relate to food choices from longitudinal studies^(15)^, and to the best of our knowledge, no such study has been conducted in Mexico. We used the Mexican Family Life Survey, a longitudinal survey, to understand how the changes in household’s income are associated with the types of food the household purchase, among all and across urban/rural area and SES.

## Methods and materials

### Participants

The Mexican Family Life Survey is a longitudinal, multi-thematic survey representative of the Mexican population at the national, urban, rural and regional levels. The Mexican Family Life Survey collects socio-economic and demographic information at the individual, household and community levels. The baseline survey followed a probabilistic, stratified and multi-staged sampling design. The purpose of the survey is to provide data to study the well-being of the Mexican population and its transitions over time^(16–18)^.

Our analysis was performed at the household level, and we used the three waves of data collection available: 2002, 2005–2006 and 2009–2012. The first survey wave collected information on 35 000 individuals in 8400 households from 150 localities. The follow-up rate for the second and third waves was close to 90 % of the original sample (around 7 % of the households were not contacted in the second wave but returned for the third). Plus, about 900 new households entered in the second wave. These new households are derived from household members of the original panel that left their home to form a new one (e.g. a son gets married). In our analysis, the new households that were derived from household members of the original panel were treated as completely new and independent households. Moreover, our analysis was limited to households that participated in at least two waves of the survey (∼8800). We excluded those that had incomplete information in any of the variables of interest or extreme values in the income variable (equal to zero, below percentile 1 or above percentile 99 of the income distribution). Thus, our final sample size was 6080 households (52 % had two waves of information and 48 % had three waves).

### Food purchases, income and covariates

Most of the data included in our analysis were reported at the household level by one of the members ≥ 18 years old (food purchases, income, household composition and education level of each member), and some were reported at the individual level by each individual ≥ 15 years old (chronic disease status).

Participants were asked about household’s purchases during the past week for thirty-three common food items (e.g. onions, potatoes, bananas, apple, rice, corn tortilla, beans, animal products milk, oil, sugar, cookies and soft drinks). For a complete list of the thiry-three food items see the tables in the results section. Information about the money spent and the value of the amount received as a gift, payment and other sources was collected for all food items. The volume purchased (kg, lt, pieces or other units) was collected for only eight food items. Thus, because we did not have the volume purchased for all food items (and in the ones available, some were reported in pieces or other units), we categorised all items into a binary variable as either purchased or not in the previous week. We did not consider items obtained through gifts, payments or other sources.

Participants were asked approximately how much each household member ≥ 5 years old earned from his job or activity to help with household expenditures during the last 12 months. We added up all the household members’ income and divided it by the number of household members to obtain income/per capita. We accounted for the effects of inflation on purchasing power with a deflation factor based on the consumers price index^(19)^.

As covariates, we included household composition, education level and chronic disease status because these variables could influence both food purchases and income. The household composition was coded with a set of four variables that included each the number of household members that were children, adolescents/young adults, female adults or male adults. Education level was coded with four variables, each with the number of household members ≥ 25 years old in each educational level (primary school or less, secondary school, high school, professional and higher). Chronic disease status was coded with six variables each with the number of household members ≥ 15 years old that reported being diagnosed with each chronic disease (diabetes, hypertension, heart disease, cancer, arthritis/rheumatism and and gastric ulcer). All of these covariates were measured in each wave and were included in the analysis as time-varying covariates.

We conducted two stratified analyses. The first by area of residence at baseline (urban/rural), with rural areas defined as populations with < 2500 habitants. The second by baseline income level was categorised as low, medium and high by tertiles of income per capita at the first available measurement.

### Statistical analyses

We first conducted a descriptive analysis and estimated the means and proportions of all variables considered in the analysis by the wave of the study (2002, 2005–2006 and 2009–2012). To estimate the longitudinal association between change in income and food purchases we conducted fixed-effects logistic models separately for each food item. The probability of purchasing the food was the dependent variable and income was the main independent variable. To estimate the effect of a meaningful change in income, we looked at the effect of one sd of the log-transformed income. We adjusted the models by time-varying household composition, education level and chronic disease status.

Fixed-effects models look at how within-household changes in income relate to within-household changes in the probability of food purchases. Because the model looks at within-household changes, each household serves as its own comparison point, and therefore all time-invariant variables are adjusted for. We conducted a fixed-effects conditional logistic model (it is conditional because all households that have all zero or all positive outcomes are dropped from the model) on the overall sample and stratified by area of residence (urban/rural) and baseline income (tertiles).

Additionally, as a comparison point and to identify the role of controlling for time-invariant unmeasured variables, we conducted a random-effects model. This model looks at both within- and between-household changes effects combined, while accounting for the clustering at the household level of the repeated observations in the estimation of the sd ^(20)^. All analyses were conducted in STATA 14 (StataCorp).

## Results

### Household’s characteristics

The mean number of household members ≥ 25 years with primary school or less was 1·2, whereas for the professional educational level of higher it was only 0·2. The number of members with at least professional education was the highest in 2009, perhaps due to the ageing of the household members. The mean number of household members ≥ 15 years that reported being diagnosed with a chronic disease ranged from 0·01 to 0·28; the number was higher for diabetes and hypertension (0·12 to 0·28) and lower for cancer (0·01) compared with other chronic diseases. The number of members with diabetes was the highest in 2009, whereas for arthritis/rheumatism it was the lowest. As expected in panel data, the mean number of young children was the lowest in 2009. The mean number of household members was 4·5–4·8. Median annual income per capita was $12 000 in wave 2002; $13 000 in wave 2005–2006 and $11 000 in wave 2009–2012 (Table 1).

**Table 1.** Household’s characteristics^*^ by the wave of the study

	2002		2005–2006		2009–2012	
	Mean	sd	Mean	sd	Mean	sd
*n*	5117		5408		4544	
Education level, number of household members ≥ 25 years						
Primary school or less	1·21	1·03	1·20	1·05	1·21	1·10
Secondary school	0·48	0·69	0·52	0·73	0·62	0·80
High-school	0·21	0·49	0·23	0·51	0·31	0·59
Professional or higher	0·17	0·47	0·19	0·51	0·23	0·59
Chronic disease status, number of household members ≥ 15 years						
Diabetes	0·12	0·35	0·14	0·38	0·20	0·44
Hypertension	0·22	0·47	0·20	0·45	0·28	0·53
Heart disease	0·05	0·22	0·04	0·21	0·05	0·22
Cancer	0·01	0·12	0·01	0·11	0·01	0·12
Arthritis/rheumatism	0·10	0·33	0·07	0·27	0·05	0·24
Gastric ulcer	0·17	0·42	0·11	0·35	0·15	0·39
Household composition, number of household members						
Children, 0–12 years	1·36	1·33	1·21	1·24	1·13	1·23
Adolescents and young adults, 13–25 years	1·17	1·22	1·25	1·28	1·34	1·31
Female adults, ≥ 26 years	1·04	0·59	1·09	0·62	1·2	0·66
Male adults ≥ 26 years	0·96	0·57	1	0·61	1·11	0·67
Household size, total number of household members	4·54	1·98	4·55	2·08	4·78	2·22
	Median	Percentile 25, Percentile 75	Median	Percentile 25, Percentile 75	Median	Percentile 25, Percentile 75
Annual income^†^, MX peso/capita	11 971	6309, 22 176	12 810	6505, 22 683	11 284	5939, 20 537

* Numbers are mean (sd).

† Inflation accounted for with the consumer price index; numbers are median (percentile 25, percentile 75).

### Changes in household’s food purchases

Overall, we did not observe large changes in the percentage of purchases between waves. The food items that were bought by ≥ 75 % of the households in all waves were onions, potatoes, chiles, tomatoes, other vegetables, corn tortillas, eggs and vegetable oil. The food items that were bought by ≤ 50 % of the households in all waves were apples, other legumes, canned tuna/sardines, fish/shellfish, cookies (crackers or sweet) and other discretionary packaged products (pastries, candies and potato chips). A similar percentage of households bought soft drinks (63–67 %) and pasteurised milk (65–71 %) (Table 2).

**Table 2.** Percentage of households that purchased the food item in the past week by the wave of the study

	2002	2005–2006	2009–2012
Onions	85	88	89
Potatoes	76	78	77
Chiles	79	79	80
Tomatoes	86	85	88
Other vegetables (lettuce, carrot, avocado and nopales)	77	78	79
Bananas	66	66	66
Apples	46	47	50
Oranges	53	44	37
Other fruits (mandarin, lemons, grapefruit and peaches)	68	66	62
Pasta soup	69	72	74
Rice	73	74	75
Corn tortilla	80	86	83
Bread from bakery	55	52	57
Other grains (bread loaf, flour, cornflakes and corn masa)	65	49	50
Beans	73	72	73
Other legumes (chickpea, lentil and fava bean)	30	28	31
Eggs	78	80	81
Chicken	70	73	73
Beef	66	69	71
Pork	32	46	51
Other animal products (lard, ham and sausage)	61	51	55
Canned tuna/sardines	42	41	36
Fish/shellfish	22	24	27
Cheese (fresh, oaxaca)	72	73	74
Pasteurised milk	65	72	71
Other dairy products (powder milk, butter and cream)	48	55	55
Vegetable oil	77	79	82
Sugar	67	77	78
Cookies (crackers or sweet)	46	45	50
Other discretionary packaged products (pastries, candies and potato chips)	37	35	27
Coffee	44	48	56
Soft drinks	67	63	63
Other beverages (juices, bottled water, alcoholic beverages and powder to flavour water)	72	72	73

### Association between change in income and food purchases

We present the results from the logistic regression models looking at the effect of a change in 1 sd in log-income/per capita on the odds of purchasing a specific food item in the last week (Table 3). The first column of results is from the random-effects model, and the second column is from the fixed-effects model. In the random-effects model, we found that an increase in 1 sd in income was associated with increased odds of purchasing most of the food items, except pasta soup, vegetable oils and sugar which had a negative association, and onions, chiles, tomatoes, rice, bread from a bakery, beans, other legumes and coffee which had a null association. In contrast, in the fixed-effects model, we found that an increase in 1 sd in income was associated with increased odds of purchasing only other fruits, beef, canned tuna/sardines, fish/shellfish, other discretionary packaged products (pastries, candies and potato chips) and soft drinks.

**Table 3.** Association between change in income^*^ and household food purchases^†^ by food items according to longitudinal logistic regression models^‡^

	Random-effects model		Fixed-effects model	
	OR	95 % CI	OR	95 % CI
Onions	0·95	0·89, 1·01	0·94	0·86, 1·03
Potatoes	**1·08**	**1·02, 1·13**	1·01	0·94, 1·08
Chiles	0·98	0·93, 1·04	1·05	0·97, 1·13
Tomatoes	0·95	0·9, 1·01	0·98	0·9, 1·07
Other vegetables (lettuce, carrot, avocado and nopales)	**1·16**	**1·11, 1·22**	1·05	0·97, 1·13
Bananas	**1·07**	**1·02, 1·12**	0·97	0·91, 1·03
Apples	**1·20**	**1·15, 1·26**	1·04	0·98, 1·11
Oranges	**1·15**	**1·11, 1·21**	1·03	0·97, 1·09
Other fruits (mandarin, lemons, grapefruit and peaches)	**1·26**	**1·21, 1·32**	**1·09**	**1·02, 1·16**
Pasta soup	**0·92**	**0·88, 0·96**	0·95	0·89, 1·02
Rice	1·00	0·95, 1·05	1·01	0·94, 1·08
Corn tortilla	**1·46**	**1·36, 1·57**	0·96	0·88, 1·06
Bread from bakery	0·97	0·92, 1·01	0·99	0·93, 1·05
Other grains (bread loaf, flour, cornflakes and corn masa)	**1·11**	**1·07, 1·16**	1·00	0·94, 1·06
Beans	1·03	0·99, 1·09	1·02	0·96, 1·1
Other legumes (chickpea, lentil and fava bean)	1·02	0·97, 1·07	0·96	0·9, 1·03
Eggs	**1·14**	**1·08, 1·2**	1·04	0·96, 1·12
Chicken	**1·09**	**1·04, 1·15**	1·00	0·94, 1·08
Beef	**1·36**	**1·29, 1·43**	**1·11**	**1·04, 1·18**
Pork	**1·13**	**1·08, 1·18**	1·03	0·97, 1·1
Other animal products (lard, ham and sausage)	**1·25**	**1·20, 1·30**	1·05	0·98, 1·11
Canned tuna/sardines	**1·23**	**1·18, 1·29**	**1·06**	**1·00, 1·13**
Fish/shellfish	**1·27**	**1·2, 1·34**	**1·09**	**1·02, 1·18**
Cheese (fresh, oaxaca)	**1·19**	**1·14, 1·25**	1·06	0·99, 1·14
Pasteurised milk	**1·37**	**1·3, 1·44**	1·03	0·96, 1·11
Other dairy products (powder milk, butter and cream)	**1·22**	**1·17, 1·27**	0·99	0·93, 1·06
Vegetable oil	**0·94**	**0·9, 0·99**	0·96	0·9, 1·04
Sugar	**0·91**	**0·87, 0·95**	0·98	0·92, 1·05
Cookies (crackers or sweet)	**1·07**	**1·03, 1·12**	1·01	0·96, 1·08
Other discretionary packaged products (pastries, candies and potato chips)	**1·16**	**1·11, 1·21**	**1·08**	**1·02, 1·16**
Coffee	1·02	0·98, 1·07	1·04	0·97, 1·1
Soft drinks	**1·23**	**1·17, 1·28**	**1·15**	**1·08, 1·23**
Other beverages (juices, bottled water, alcoholic beverages and powder to flavour water)	**1·35**	**1·28, 1·42**	1·07	0·99, 1·15

Numbers in bold are statistically signficant (*P* < 0·05).

* 1 sd change in the natural log of income per capita.

† Probability of purchasing during the last week.

‡ Both models were adjusted by time-varying education level, household composition and chronic disease status.

**Figure 1.**
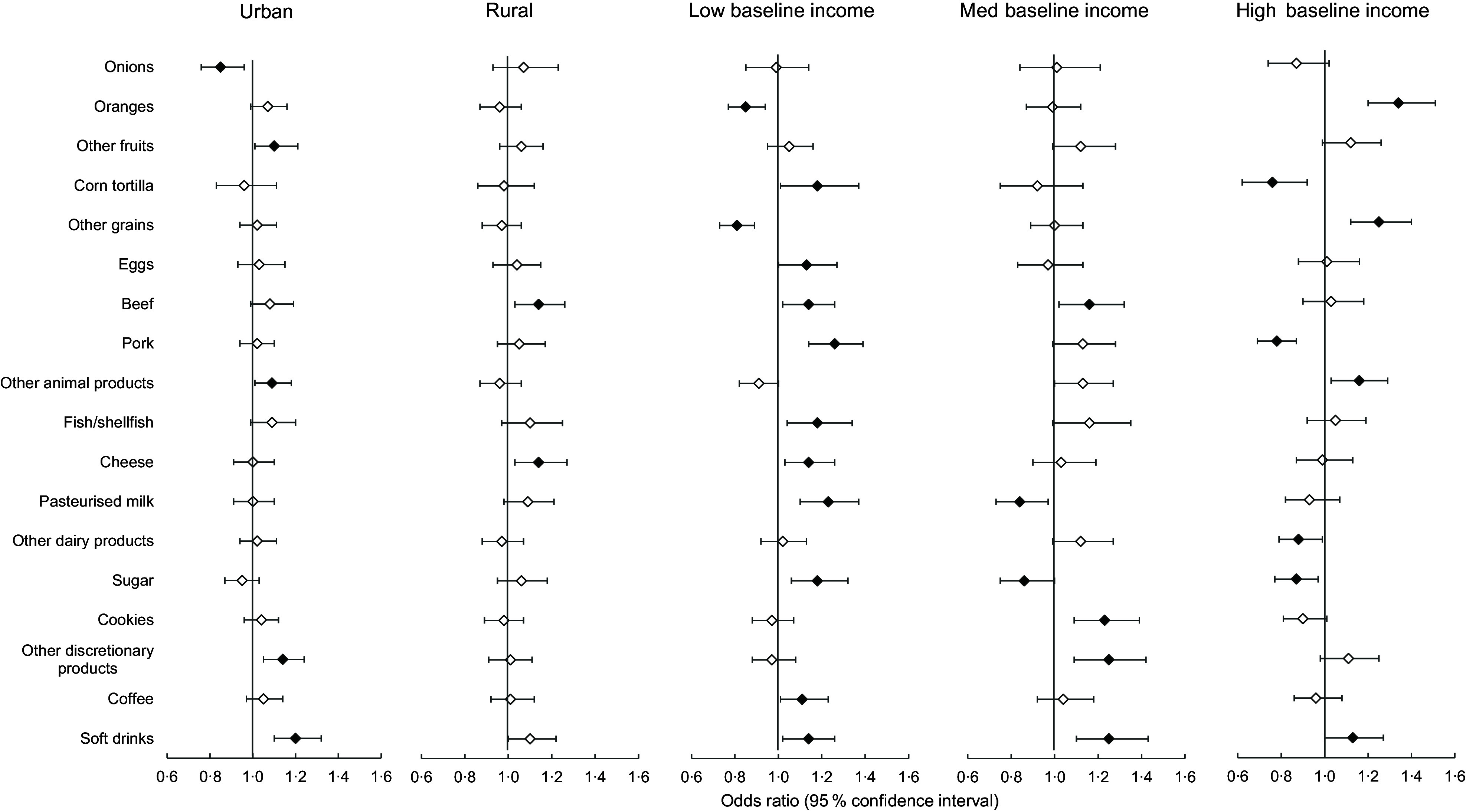
Association between income increase^a^ and household food purchases^b^ by area of residence and baseline income levels^c^. ^a^1 sd change in the natural log of income/per capita. ^b^Probability of purchasing during the last week. ^c^Fixed-effects models adjusted by time-varying education level, household composition and chronic disease status. Only foods with at least one statistically significant result are presented. Data points in black are stastistically significant (*P* < 0·05).

We present fixed-effects analysis stratified by urban/rural areas and tertiles of baseline income (only foods with at least one statistically significant result are presented) (Figure 1). For the urban stratum, an increase in income was associated with lower odds of purchasing onions and increased odds of purchasing other fruits (mandarin, lemons, grapefruit and peaches), other animal products (lard, ham and sausage), other discretionary packaged products (pastries, candies and potato chips) and soft drinks, whereas for the rural strata, it was associated with increased odds of purchasing beef and cheese. For the low baseline income category, an increase of 1 sd in log/income per capita was associated with lower odds of purchasing oranges and other grains (bread loaf, flour, corn flakes andcorn masa) and increased odds of purchasing corn tortillas, eggs, beef, pork, cheese, milk, other dairy products, sugar, coffee and soft drinks. An increase in income for the medium baseline income category was associated with decreased odds of purchasing milk and sugar and increased odds of purchasing beef, cookies, other discretionary packaged products and soft drinks, whereas in the high baseline income category, it was associated with lower odds of purchasing corn tortillas, pork, dairy products other than milk and cheese and sugar and increased odds of purchasing oranges, other grains and soft drinks.

## Discussion

We used data from three waves of the Mexican Family Life Survey (2002, 2005–2006, 2009–2012) to evaluate the association between changes in income and the type of food purchases at the household level. Our hypothesis was confirmed, as income change was positively associated with a mix of items in terms of healthfulness and sustainability, with variations by urban/rural and baseline income. Income increase was associated with the odds of purchasing healthy items such as other fruits, canned tuna/sardines and fish/shellfish; unhealthy items such as other discretionary packaged products (pastries, candies and potato chips) and soft drinks and unsustainable items such as beef. Findings varied by urban/rural area or baseline income. Yet, even if not always statistically significant, the odds of purchasing other fruits, beef, fish/shellfish and soft drinks were higher with higher income across all stratum. Also, many more items were positively associated with increased income among those with low baseline income and very few were associated among those living in rural areas.

The association between income and the demand for a good is defined as income elasticity. Normal goods have positive income elasticities which means that the demand increases as income increases, while inferior goods have a negative elasticity. In our analysis, we were not able to estimate income elasticity per se, as we only analyzed the probability of purchasing and not the amount purchased. If we assume that probability and amount tend to be correlated, we could interpret from our results, based on the fixed-effects model that other fruits, beef, canned tuna/sardines, fish/shellfish, other discretionary packaged products (pastries, candies and potato chips) and soft drinks are normal goods (either necessity or luxury goods) in the Mexican population. In the fixed-effects model, none of the foods was identified as an inferior good; however, based on the random-effects model, pasta soup and vegetable oil could be considered inferior. Our results are in line with other studies from low- and middle-income countries. A meta-analysis from Africa reported that the foods with highest positive income elasticities were beverages and animal-based foods^(21)^, while a study in Guatemala found that beverages, including sof had positive expenditure elasticities which implies they are normal goods^(22)^.

In this study, we found many more associations with the random effects than with the fixed-effects models. It is known that random-effects models tend to give more precise results than fixed-effects models because these models incorporate all the information available in the model (both within- and between-household differences). However, random-effects models could be biased. Income could be associated with many household characteristics that might remain invariant even when income changes (e.g. culinary traditions, family background, food preferences, city/neighbourhood/school/work food environment and health behaviour and knowledge) and these are controlled for in a fixed-effects model (assuming these are time invariant).

The food sources and the type of stores available play an important role in food purchasing decisions^(23–25)^ and might be one of these such factors that are strongly associated with income, but that might not change much when income increases (e.g. households remain in the same neighbourhood). In this study, we found that the food purchases among the rural population remained relatively stable, only increasing the likelihood of purchasing beef and cheese (two out of thirty-three food groups) after an increase in income. This may be related to rural households having fewer options to purchase foods compared with urban households^(26,27)^ and may also rely more on locally sourced foods, produced in their household or nearby areas.

We found that households with the lower baseline income level were the most susceptible to modifying the types of foods purchased, twelve out of thirty-three food groups changed (ten increased and two decreased). In contrast, among households with high baseline income eight food groups changed (four increased, four decreased). This is consistent with findings from Brazil that report a smaller income effect on the purchase of foods in high-income households compared with lower-income households^(28)^. Moreover, the emphasis on purchasing many animal-based foods, such as eggs, beef, pork, fish/shellfish, cheese and milk, among those with lower baseline income is consistent with the purchase/intake pattern observed previously in higher SES^(29–31)^. Low-income households tend to purchase more inexpensive grain-based staple foods that are energy-dense such as corn-based products^(32)^. In this study, among those with lower baseline income, the likelihood of purchasing corn tortillas increased while other grains (such as bread loaf, flour, cornflakes and corn masa) decreased after an increase in income. It is possible that the other grains were mainly corn masa or flour, which is cheaper than corn tortillas. On the contrary, among those with higher baseline income, the likelihood of purchasing corn tortillas decreased and other grains increased. In this case, likely the other grains were mainly bread loaf, which is more expensive than corn tortillas.

We found that in terms of healthfulness and/or sustainability, the effect of income was mixed; on the positive side, other fruits and fish/shellfish increased, and on the negative side, beef, soft drinks and processed discretionary foods also increased. Beef is the animal food source that has the most environmental impact. Greenhouse gas emissions by a gram of protein of beef are ∼7 times greater than those of other animal sources and 20–50 times greater than those of legumes, nuts and seeds^(33)^. Hence, our findings are worrisome, as we found that beef was one of the items most consistently associated with income across urban/rural areas and income levels. Moreover, we found that the lower baseline income households give preference to the purchase of animal-based foods over fruits and vegetables. Soft drinks also increased with increased income consistently across subpopulations. This reflects the widespread demand for these industrialised beverages regardless of SES, as well as its ubiquitous presence among all types of stores and food outlets. The Mexican government implemented a tax on sugar-sweetened beverages in 2014, successfully reducing its intake/purchase, particularly among lower-income households^(34)^. This confirms that the purchase of soft drinks is strongly related to households’ purchasing power. A higher tax could potentially broaden its effects and achieve similar results among higher-income groups.

In this study, we modelled the possible effect of income changes, as a continuous variable, on food purchases. We presented the results of income increases, but likewise with our results we could identify and interpret the effect of income decreases, which would be the opposite of our findings. This means that if income decreases, the purchases of fruits, fish/shellfish, beef, soft drinks and processed discretionary foods would decrease. Again, this implies a mixed effect on diet quality and sustainability; with decreases in both healthy and unhealthy foods.

There are important limitations to our study. First, the food purchase data were not detailed. For instance, we were not able to identify changes in the amount of purchased foods and limited our analysis to changes in the probability of purchase. Yet, probability and amount tend to be correlated, and usually, the probability contributes more than the amount to dietary intake estimations^(35,36)^. As an example, the effect of taxes on the purchases of foods in Mexico was larger in the probability than in the amount^(37)^. In addition, the number of food items queried was extensive, but some items such as *other grains* and *other beverages* were too broad to be able to differentiate healthy/unhealthy products within the item. A second limitation is that the data are from more than 10 years ago, and although the food preferences, environment and policies are continuously shifting, the changes are small (as evidenced in the 7-year window of our study), and we do not expect our results to differ dramatically with more recent data. Among the strengths of this study is the use of longitudinal data from a representative sample of Mexican households. By using fixed-effects models, we were able to look at within-household change over the repeated measures and control for time-invariant characteristics plus we control for important time-varying characteristics such as household composition, education level and chronic diseases.

These results suggest that if the household’s income of Mexicans grows, we could expect mixed results on the healthfulness and sustainability of food purchases. On one hand, the purchases of healthy and/or sustainable foods such as other fruits, canned tuna/sardines and fish/shellfish would increase. But, on the other hand, the purchases of unhealthy and/or unsustainable foods such as beef, other discretionary packaged products (pastries, candies and potato chips) and soft drinks would increase also. Therefore, these results highlight the importance of having public policies aimed at improving the food environment and delivering nutrition education to the population to enhance the positive effects and counteract the negative effects of income on food purchases decisions. Also, economic crisis or income growth is important variables that should be considered when evaluating the effect of these public policies.
